# Hemorrhoids and the General Surgeon

**Published:** 1916-08

**Authors:** Rollin H. Barnes

**Affiliations:** Editor of The Proctologist and Gastroenterologist. Fellow of the American Proctologic Society. St. Louis, Missouri


### Hemorrhoids and the General Surgeon

ROLLIN H. BARNES, M. D.,

Editor of The Proctologist and Gastroenterologist.

Fellow of the American Proctologic Society.

St. Louis, Missouri.

It seems peculiar that the general man who is so full of surgical procedure should when it comes to the hemorrhoidal region forget all this knowledge and use methods so obsolete, in this particular region.

Hemorrhoids are tumors the result of dilatation of the hemorrhoidal plexus of veins. This condition known since the most ancient history has not an accepted pathology, so I suppose one has as much right to theorize as another. Why should we look for the most complicated pathology when a simple one is more reasonable and more apt to be correct? I consider the dilatation of the veins of this plexus to be due to some inflammatory process that weakens the mucous membrane overlying them. They gradually enlarge and inflammatory processes result in the well developed hemorrhoid.

Some have considered hemorrhoids a very minor trouble. It is no doubt that they produce considerable suffering and, long continued inflammation in this region of abundant lymphatics, results in toxic absorption that produces systemic troubles, the extent of which we are just beginning to recognize. Physicians generally have been led to believe that hemorrhoids amount to little, and they allow these patients with hemorrhoids, without proper advice, to suffer for years until they end in some serious condition. When a patient complains of trouble in this region it is very important that the attending physician should go into careful diagnosis of the existing condition and carry out the proper treatment for I consider this to be along the line of preventative medicine.

If these cases are seen early there is no question but what thorough treatment of the case will prevent extensive hemorrhoidal development and it will not always be necessary to perform operative treatment. When once inflammatory processes have resulted in well developed hemorrhoids, no delay should be taken in their removal by operative methods.

There are many methods in use that are obsolete and not in accord with our surgical knowledge of today. I cannot understand why one who is trained in surgery would think of injecting phenol into a tumor mass, in order that it may slough away. Equally obsolete is the tying of a ligature around a hemorrhoid or the cauterizing with red hot iron that will result in a slough. Is it not more reasonable to remove these tumors by a clean cut longitudinal eliptical incision so that when the parts contract the edges of the wound will coapt evenly? There has been much fear and exaggeration of the danger of hemorrhage from such a procedure, but the day is past when a surgeon should not be able to control a primary hemorrhage the result of an incision that involves the smaller blood vessels. No one has seen extensive hemorrhage from the anal canal when the sphincter contraction is normal. We should avoid such methods as would lead to relaxation of these parts. I do not think it is good surgery to use sutures in this region unless it is absolutely necessary because it is impossible to thoroughly sterilize this region. There is always infection present and we know that a suture will carry infection into the tissue. When a suture is used additional tissue is involved, hence I believe in the open method for the removal of hemorrhoids without the use of sutures.

---

**Ochronosis and Melanuria.** B. S. Oppenheimer and N. W. Janney, Med. Rec., May 6. The condition was first named and described by Virchow, 50 years ago, the name being peculiarly inappropriate (ochros, pale, wan, yellow, yolk of egg). Albrecht 1902, noted the occurrence of alkaptonuria. Ludwig Pick, 1906, described a group of cases due to phenol poisoning. The patient was a male, aged 40, without significant family history—no consanguineous marriages—denying venereal disease. He weighed 104 pounds on admission, had cough, occasional haemoptysis, obstinate constipation and complained of spinal pains, Geo. R. Elliott finding ankylosis of lower 3/4 of vertebral column, an osteoarthritis the primary lesion being degeneration of the intervertebral cartilages. Typic pigment deposits were noted, the entire eyeball except cornea becoming brownish-black within half an hour after death. A loud systolic murmur, thickened arteries and enlarged liver were also noted. Melanogen was present in the urine, which darkened on exposure to light. In this case, alcaptonuria was excluded, there being no homogentesic acid. The melanin present contained C H O N S and Fe. Ochronosis may be divided into three classes: those due to phenol poisoning, those in which alcapton or a modification is present, those like the present, in which a similar ferment action occurs on proteids containing an oxy-phenyl radicle.

